# “I Can Remember Sort of Vivid People…but to Me They Were Plasticine.” Delusions on the Intensive Care Unit: What Do Patients Think Is Going On?

**DOI:** 10.1371/journal.pone.0153775

**Published:** 2016-04-20

**Authors:** Julie L. Darbyshire, Paul R. Greig, Sarah Vollam, J. Duncan Young, Lisa Hinton

**Affiliations:** 1 Nuffield Department of Clinical Neurosciences, University of Oxford, Oxford, United Kingdom; 2 Oxford University Hospitals National Health Service Foundation Trust, Oxford, United Kingdom; 3 Department of Primary Care, University of Oxford, Oxford, United Kingdom; Shinshu University School of Medicine, JAPAN

## Abstract

**Introduction:**

Patients who develop intensive care unit (ICU) acquired delirium stay longer in the ICU, and hospital, and are at risk of long-term mental and physical health problems. Despite guidelines for patient assessment, risk limitation, and treatment in the ICU population, delirium and associated delusions remain a relatively common occurrence on the ICU. There is considerable information in the literature describing the incidence, suspected causes of, and discussion of the benefits and side-effects of the various treatments for delirium in the ICU. But peer-reviewed patient-focused research is almost non-existent. There is therefore a very limited understanding of the reality of delusions in the intensive care unit from the patient’s point of view.

**Method:**

A secondary analysis of the original interviews conducted by the University of Oxford Health Experiences Research Group was undertaken to explore themes relating specifically to sleep and delirium.

**Results:**

Patients describe a liminal existence on the ICU. On the threshold of consciousness their reality is uncertain and their sense of self is exposed. Lack of autonomy in an unfamiliar environment prompts patients to develop explanations and understandings for themselves with no foothold in fact.

**Conclusion:**

Patients on the ICU are perhaps more disoriented than they appear and early psychological intervention in the form of repeated orientation whilst in the ICU might improve the patient experience and defend against development of side-effects.

## Introduction

Delirium in patients on the intensive care unit is recognised as an unwelcome consequence of the experience and environment. Patients who become delirious stay longer in the intensive care unit (ICU) and hospital [1], and are at risk of long-term mental and physical health problems including debilitative cognitive degradation [2–4]. In addition to being an independent risk factor for more serious physical morbidities and mortality [5–7], this represents an on-going burden to the patients, their carers, and to the National Health Service [8, 9].

Although the pathophysiology of ICU-acquired delirium is poorly understood, it is commonly considered to be related to disturbances in neurotransmission, either due to a natural response to severe illness, or as a result of drug induced changes [10–17]. Delirium may manifest in increased agitation or activity, paranoid thoughts, hallucinations, or the patient may become withdrawn and disinterested in communication. It can be hard to identify these hypo-delirious individuals in the ICU because many patients are partially sedated and/or can find it difficult or impossible to speak. In all cases patients are at risk of self-injury as a result of dislodging equipment or falling out of bed in an attempt to escape. There are clear benefits to recognising delirium early, reducing its duration, and limiting its enduring effects [18].

Delirium is characterised by a rapid change in mental state, detectable through validated screening tools such as the Confusion Assessment Method for the ICU (CAM-ICU). Current guidelines recommend screening all patients at least daily [19] but there are obvious limitations for sedated/ventilated patients and the tool may be less sensitive for those patients with hypoactive delirium [20–22].

Estimates suggest between 30–75% of all patients experience at least one delirious episode whilst they are in the ICU [1, 23–25] and there is evidence that it is often not recognised [2]. In the UK, the first line of treatment for hyperactive delirium is haloperidol [26]. Other pharmacological treatments include benzodiazepines, propofol and atypical antipsychotics. Some of these agents are themselves potentially deliriogenic [27–30].

There is a large body of medical literature that describes the incidence [1, 23–25] and suspected causes of delirium in the ICU [10–17, 31, 32], along with discussion of the benefits and side-effects of treatment. There is however limited understanding of the reality of the delusions that can occur in the context of delirium as experienced by the patient in the ICU. Descriptors of psychosis are largely limited to the psychiatric patient population [33, 34], and drug induced symptoms for agents including ketamine [35], cannabis [36], cocaine [37–41], and LSD [42, 43]. Psychotic features of Parkinson’s disease have also been described [44]. There are detailed descriptions of the nature of ICU-delirium and associated delusionary thoughts and hallucinations within the realms of public and social media [45–49], but published, peer-reviewed research is rare.

This paper sets out to describe the patients’ experiences in their own words, based on a qualitative secondary analysis of narrative interviews with patients treated on UK ICUs in 2005–6. The interview data presented here are patients’ own reflections and recollections of their experiences and there is no clinical link made to their intensive care diagnoses or treatments. While there are obvious limitations to this, the patients’ descriptions of their intensive care experiences are rarely reported and remain a valid and useful insight into their understanding of the intensive care environment.

## Methods

### Setting

The original study collected patient and relatives’ experiences of treatment on intensive care. Participants’ experiences of the ICU are from between 1994 and 2005. A maximum variation sample was recruited and included both men and women who had been admitted to the ICU as emergency and elective cases, as well as those who were cared for on units for different durations. See Table 1. Participants were approached to take part through health professionals, charities, and support groups. In-depth interviews were recorded in 2005–6 around the UK, and results of the original analysis were published on the Healthtalk website in 2006 and in the scientific press [50, 51]. The original study was granted ethical approval from the Eastern MREC (ref: 03/5/016) and all patients consented to take part in the ‘personal experiences of health and illness project’. Where patients consent to future use of their interviews for education and research, Healthtalk project interviews are copyrighted and archived at the University of Oxford. For this study, access to the archived interviews was granted by the Health Experiences Research Group (HERG), the custodians of the archive, to allow a secondary analysis of anonymised transcripts exploring themes relating to sleep and delirium.

**Table 1:** Patient demographics.

Patient ID	Age at interview	Sex	Admission	Year of ICU admission	Length of ICU stay	Length of HDU stay	Length of post-ICU ward stay	ICU follow up
**1**	41	F	Emergency	1998	11 day	None or unknown	2 weeks	Regular appointments with clinical psychologist
**2**	60	M	Emergency	2004	about 1 month	None or unknown	4 days	Attended initial appointments, declined future appointments
**3**	66	M	Emergency	2002	5 weeks	None or unknown	5 weeks	1 appointment
**4**	46	F	Emergency	Not reported	about 5 weeks	1 week	Discharged after 1 week in HDU	2 appointments
**5**	40	F	Emergency	Not reported	22 days	10 days	Just over a week	1 appointment
**6**	35	F	Emergency	2003	3 weeks	36 hours	5 weeks	2 appointments and private counselling
**7**	60	M	Emergency	2004	7 months	None or unknown	1 month	None at time of interview
**8**	50	M	Emergency	2004	10 days	None or unknown	Readmitted in 2006 and died	Not reported
**9**	60	M	Emergency	2004	3 days	None or unknown	10 days	No ICU follow-up
**10**	76	F	Emergency	2004	about 1 week	None or unknown	1 week	1 appointment
**11**	37	F	Emergency	2002	Unknown duration	None or unknown	Not reported	Not reported
**12**	23	F	Emergency	2005	21 days	2 weeks	2 weeks	Had been invited to attend 1st appointment
**13**	42	F	Elective Surgery	1998	Not reported	None or unknown	Not reported	Not reported
**14**	35	F	Emergency	2005	2 days	None or unknown	2 days, discharged herself	Declined appointment
**15**	38	F	Emergency	2004	1 month, admitted three times in 2004	None or unknown	Several months on and off	No ICU follow-up
**16**	67	M	Emergency	1994	8 weeks	None or unknown	3 weeks	No ICU follow-up
**17**	30	M	Emergency	2004	12 days	None or unknown	2 weeks	No ICU follow-up
**18**	62	M	Emergency	2004	18 days	None or unknown	5 weeks	2 appointments
**19**	58	M	Elective Surgery	2004	Not reported	None or unknown	Not reported	Not reported
**20**	63	M	Elective Surgery	2004	Not reported	None or unknown	Not reported	Not reported
**21**	72	M	Emergency	2001	about 4 weeks	None or unknown	1 week	No ICU follow-up
**22**	71	M	Emergency	2004	17 days	None or unknown	A few days	At least 1 appointment
**23**	54	M	Emergency	2004	17 days	None or unknown	2 weeks	1 appointment
**24**	44	F	Emergency	2004	5 weeks	None or unknown	2 weeks	Physiotherapy referral after 1st appointment, GP referral for counselling
**25**	45	M	Emergency	2003	4 weeks	None or unknown	2 weeks	2/3 appointments
**26**	47	M	Emergency	2005	30 days	None or unknown	8 days	No ICU follow-up
**27**	68	M	Emergency	2005	2 weeks	2 weeks	Ward: 6 weeks; Rehab: 2 weeks	1 appointment
**28**	46	M	Emergency	2004	7 weeks	None or unknown	4 weeks	1 appointment
**29**	47	F	Emergency	2004	2 weeks	1 day	1 week	1 appointment, another expected
**30**	55	F	Emergency	2005	6 days	5 days	Discharged after HDU	2 appointments
**31**	71	M	Emergency	2003	2 weeks	None or unknown	3 weeks	At least 1 appointment
**32**	57	F	Emergency	Not reported	29 days	None or unknown	Ward: 6 weeks; Rehab: 6 weeks	3 appointments
**33**	43	M	Elective Surgery	2004	Not reported	None or unknown	Not reported	Not reported
**34**	37	M	Emergency	2001	30 days total, admitted twice	None or unknown	Several months	At least 1 appointment
**35**	33	M	Emergency	2004	17 days	None or unknown	Ward: 1 week; Rehab: 3 months	At least 1 appointment
**36**	67	F	Emergency	Not reported	Could not remember	None or unknown	Not reported	Awaiting 1st appointment
**37**	58	M	Emergency	Not reported	9 days	None or unknown	16 days	At least 1 appointment
**38**	55	F	Emergency	2005	1 week	None or unknown	2 weeks	1 appointment
**39**	56	M	Emergency	2005	4 days	None or unknown	3 days	No ICU follow-up
**40**	71	M	Elective Surgery	2005	Not reported	None or unknown	Not reported	Not reported

### Design

The purpose of the original interviews was to develop a broad overview of patients’ experiences of treatment on an ICU. Secondary analysis allows exploration of a new conceptual focus using existing data [52].

The rich data source included in the database [53] enables detailed review of specific themes, and transcripts of all 77 interviews (40 patients, 37 relatives) were made available for the secondary analysis. Two researchers reviewed the text with a specific focus on sleep and delirium, and constructed a coding framework as outlined by Ziebland and McPherson [54] which was adapted as new themes emerged. We then undertook at thematic analysis of these extracts using constant comparison, seeking to define and interpret the concepts within the data [55]. During this process the richness of the data provided by patients describing their delirious experiences became apparent. In this re-contextualisation of the data [56], the core message, or the ‘body’ [57], in the results was discovered.

It is not the intent of qualitative research to offer statistically irrefutable results and, as such, does not attempt to define itself numerically. In keeping with the accepted reporting of qualitative studies this analysis makes no attempt to present numerical results as these are likely to mislead [58, 59]. Including numbers and proportions suggests representativeness, which is not the case for qualitative work. The strength of qualitative work is that it can offer rich sources of information and insight into the lived patient experience, in this case of being critically ill and treated in an ICU, which can offer a better understanding of a topic. We do not attempt to generalise, because we acknowledge we cannot do that. However, through this presentation of patient voices and perspectives we can raise awareness in the clinical community of some of the concepts that may underlie their patients’ apparent disinterest, or overt delirious or delusional actions.

## Results

We anticipated themes that would include descriptions of dreams, nightmares, and hallucinations. Themes that emerged from our secondary analysis included noise on the ICU, particularly from equipment, other patients and their visitors, personal (dis)comfort, and disturbance from treatments.

Many patients reported feelings of disorientation and separation whilst being treated on the ICU. Some patients were able to recall their dreams or hallucinations. Few truly distinguished between the two. Patients were able to give detailed descriptions of what they thought was happening to them at the time. Although it is possible to retrofit aspects of clinical care to some of the accounts, it was rare for specific details from patients’ dreams or hallucinations to directly reflect what was actually taking place. Through the interviews there is an overwhelming sense of complete bewilderment and fear expressed in nightmares, altered realities, and false explanations. People often do not internalise the rational account of what they are seeing and instead create their own stories to fit their perceived situation.

The results presented here focus on patients’ own descriptions of their emotional and sense-making experiences in the ICU including awareness of their surroundings, feelings of isolation, paranoia and blurred realities that can lead to delusional beliefs.

### Making sense of the environment

Patients find the 24-hour activity cycle, bustle, frequent disturbances, and variable light levels challenging. This could be a trigger for confusion.

“there’s so much going on around you”

[Patient 18];

“machines that have flashing lights and bells and all sorts of things like that”

[Patient 20];

*“there always seemed to be parties going on in the ward*. *When I actually saw the ward…there was about six beds in there…*.*The way I saw it*, *it was huge with an upstairs”*

[Patient 27];

*“I found it very noisy*, *very hot*, *very busy*.*”*

[Patient 38]

Many patients were unable to recognise contemporaneously that they were ill, or in hospital, which is consistent with difficulties in interpreting the (often unpleasant) treatments that patients receive whilst on the ICU. Many patients describe feelings of bewilderment and abandonment. This often manifests in the meaning they attribute to their time on the ICU. Some patients describe going through a process of “*trying to piece together what was happening*” [Patient 06] by attempting to make sense of their unfamiliar surroundings. Reflecting themes of confusion and discomfort many report beliefs suggesting travel. Some made very specific references to being on a ship, boat, train, aeroplane, or in a spaceship.

*“I thought I was on a hospital aeroplane and that I was being looked after by kind of air hostesses/nurses/doctors… it is quite hard to kind of switch off*.*”*

[Patient 06]

Some believed they had been abducted, others talked of their feelings of restraint and imprisonment.

*“I couldn’t move*, *couldn’t move at all*, *I felt my throat*, *and I knew that I had a pipe into my throat*, *a thick pipe*. *I could feel the wires*, *I could see all the wires in my arms and my legs*, *I just didn’t know what had happened*, *I was just*, *I just didn’t know*. *I just didn’t have a clue*. *And I thought*, *I thought aright I’ve been kidnapped*, *and I’m in a clinic*, *and I’m somewhere like Dubai or somewhere like that*, *but I know my family will be looking for me”*

[Patient 05];

“I was I think in India I think, I don’t really know where I was. There was, high up a mountain, there was lots of little places and there was people, oh people were looking for me, that’s right yeah, people were looking for me, so she [the nurse] was hiding me as well…I was imprisoned and just couldn’t, people were sitting on me and I just couldn’t get out, because I was restricted where I was and I couldn’t move.”

[*Patient* 07]

Even those who understood they were in hospital experienced vivid dreams (rarely did patients differentiate between dreams and hallucinations in their accounts, it is possible these were hallucinations) in which they reported similar sensations of victimisation.

*“Part of the nightmares that I’d had…was that I had been kidnapped by triads*, *don’t ask me where they came from*. *I dreamt that they cut off my little finger*, *when I woke up no matter how many times I counted them there was only four…so I was convinced that had truly happened”*

[Patient 05];

*“I’d have this nightmare about tubes and oh*, *God*, *it was crazy*. *And like I’d*, *I’d wake up thinking I was somewhere else and I don’t…it was totally weird*.*”*

[Patient 12]

Some reported actively trying to avoid sleep to escape their dreams.

*“Each time I went under*, *the nightmares flooded back*. *So when they were telling me to sleep*, *I couldn’t sleep because a blackness just come back over me*, *so I stayed awake*.*”*

[Patient 07]

The origin of some patients’ delusions seems consistent with plausible clinical events of their admission. This account for example could be linked to changes being made to ventilator settings.

*“I felt I was something like a washing machine built into a shop or something*. *And I was just plugged in to the system and people every now and again came and twiddled my*, *made adjustments here and there*.*”*

[Patient 02]

Others were more inexplicable. Some of the descriptions offered by the patients were specific but surreal, and difficult to attribute to any particular event but continue to convey a sense of loss of autonomy.

*“a person finding himself in tubes*, *metal tubes*, *ice tubes*, *not able to get out them*, *going round and round*. *And I know exactly where these tubes are … junction 22 of the M62 motorway*.*”*

[Patient 27]

Some patients feel reassured when they recognise they are in hospital. They can attribute genuine and accurate meaning to their experiences. There seems to be a release of tension as their sense of anxiety and threat is reduced.

*“once I was convinced I was in hospital I did start to feel a bit more secure*. *But until then I didn’t*.*”*

[Patient 05]

The realisation of being in ICU is not reassuring for all patients. Some explained that seeing sick patients around them forced them to reflect on their own condition. If the patients around them looked near to death then they must also be in a similar situation.

*“I’m looking round and you can see all*, *everybody looks dying so I’m thinking I’m dying here*.*”*

[Patient 28]

### Isolation

A number of them felt particularly isolated and vulnerable, mostly due to the difficulties involved in communicating their fears and discomfort to the staff caring for them.

*“I couldn’t make anybody understand*. *I couldn’t make my family understand*. *I tried to tell them*, *I couldn’t talk*.*”*

[Patient 29]

While patients were in the ICU they seemed unable to place events going on around them into context. Some expressed distress at not knowing whether the tasks around them were routine or more significant. Their inability to ask questions of their carers was also a source of difficulty. In retrospect patients felt excluded from their care.

*“you were isolated*. *They would come round and fiddle with things behind you*, *but you couldn’t see what was there and you weren’t actually told what was there*. *And for a long time of course with the tubes in my mouth I couldn’t ask”*

[Patient 30];

*“you can’t really see all of the kind of equipment which*, *you know*, *presumably because they don’t want to scare the living daylights out of you”*

[Patient 06];

*“you hear something bleeping and you think on what-what’s happening*, *but you couldn’t do anything about it*.*”*

[Patient 34]

Some tried to express how profoundly debilitating their ICU experience had been, stripping them of dignity and their ability to perform basic human tasks, like talking, eating, sleeping. Patients’ descriptions suggested that their individuality, even their humanity, was lost to them while they were in the ICU.

“I felt like a lump of meat on a butcher’s table with the real me inside but not able to get out……I was part of this and yet I wasn’t part of it”

[Patient 27];

*“I was out*, *as this fish*, *in a boat*, *a glass-bottomed boat*. *But I was in the boat and people were looking through the glass bottom at me*, *as opposed to the other way round*.*”*

[Patient 26]

### Paranoia

Many patients expressed a sense of paranoia. They attributed conversations amongst and between staff members to them and describe what in other contexts would be described as ‘persecutory delusions’ and ‘delusions of reference’ [60].

*“they were looking at something in the magazine together and they were laughing*, *and I thought they were laughing*, *that I was in the magazine and they were laughing at me*. *And I thought that I had a cat’s nose painted on and whiskers and that I was in the magazine like that that and they were laughing at me…I imagined that they were talking about me all the time*, *saying that I was a drug addict and I shouldn’t even be in there”*

[Patient 05];

*“I thought the nurses had this little bit at the front where they sat and wrote their reports and did whatever they had to do and I thought they had some sort of game that they were playing on the wall*, *making patterns*. *Obviously I mean that’s ridiculous but*, *you know*, *it’s the way you think*. *And I thought oh well that’s something to keep them occupied while they’re waiting for all these people to do what ever they’re going to do*.*”*

[Patient 02]

A number of patients expressed a sense of direct threat coming from the staff.

*“I remember one of the doctors that was on Intensive Care*, *who I really thought was trying to kill me*. *He was*, *I don’t think he actually did any treatment on me*, *but every time I saw him I was terrified*…*if he came anywhere near me*, *I thought he was trying to switch the tubes off that were feeding me or helping me to breathe or whatever…[I] was absolutely convinced it was real and would have rather gone home and died that have him touch me”*

[Patient 29];

*“the nurse … got a gun to me head trying to kill me…and I’m frightened because I think people are trying to kill me*.*”*

[Patient 28]

### Blurred reality

Several patients were able to explain that they knew the lines between reality and their understanding were blurred. Although these accounts are retrospective, patients describe being aware at the time that they were confusing their own realities with information they were seeing or hearing from televisions, or visitors. Despite this, they were still unable to fully understand the difference between fact and fiction.

*“kind of like being in a Twilight Zone it’s*, *“Have I dreamt that or did it happen*? *Or is this real*? *Or have I imagined it*?*” So you need telling not once but several times”*

[Patient 01];

*“I’m thinking*, *like we’re in the jungle and then everything was on fire*, *and everything was getting mixed up with the dreams and I was worried that the triads were coming back and the nurses were in cahoots*.*”*

[Patient 05]

Some patients only understood afterwards that their version of events was not real.

*“I thought I had a necklace on that was too tight*. *And it was only afterwards I realised it was because it was a tube that I was breathing through…and I couldn’t understand why she was letting me sleep with a necklace that was so tight”*

[Patient 29];

*“I can remember sort of vivid people…but to me they were plasticine*. *They weren’t real*. *But obviously they were real because they were obviously talking to me*. *So the words registered but the mind didn’t take in that they were actually real people*. *They were like Wallace and Gromit type characters*.*”*

[Patient 23]

Even apparently innocuous stimuli were sufficient to provoke abnormal thoughts or distress amongst some of the patients.

*“I’d wake up in the night and I’d look at things and think it was something entirely else*, *you know*. *We had a balloon*, *one of the balloons*, *Get Well balloons which we had up*. *I could see that and as far as I was concerned that was a Bogey Man or whatever*, *you know what they call that*, *that was floating around and that in the middle of the night*, *that really worried me”*

[Patient 07];

*“Lights play tricks on the mind*, *so that in the middle of the night when the lights are on in the ward*, *lights suddenly become faces*, *which are quite scary*.*”*

[Patient 27]

Many patients who were able to describe their “mad bizarre dreams” [Patient 05] were aware, with hindsight, that these were a result of either their illness, or the medications used to treat it.

*“I don’t know it at the time but they put that many drugs in to me that I’m tripping*, *like hallucinating things and I don’t know what’s what*.*”*

[Patient 28]

However, this realisation after the event can have no impact on the reality of very real fear at the time. In fact some patients felt that too much emphasis was placed on trying to convince them that their fears were not real.

*“I felt they should have understood that from my point of view it was true*, *and nobody was going to convince me otherwise*. *And it still feels true*. *Although I know now with hindsight it can’t have been*, *it still feels true to me when I think about it*.*”*

[Patient 29]

## Discussion

Delirium and delusionary experiences have been recognised for thousands of years and it is more common in the contemporary ICU than many realise. Previous research has focussed on the staff experience of delirious patients, including aetiology, treatment, and diagnosis. This may be a result of an emphasis on treatment for patients with hyperactive delirious symptoms who may present an overt danger to themselves and staff. This study uses the rich data provided by the patients themselves to paint a vivid picture of what the ICU can be like from their perspective. This previously unreported patient perspective demonstrates that whilst no two patients’ are identical, there are similarities in both the detail and underlying beliefs. This makes it possible to contemplate ways in which the patient experience of the ICU could be improved.

Even when patients recognised they were seriously ill, they did not necessarily associate this with being in hospital. The general public is unlikely to have a great deal of exposure to a real ICU environment. Media portrayals seldom closely depict the reality of day-to-day clinical practice. For many patients, their admission to ICU is often unexpected and far removed from even a routine hospital admission, where patients tend to be fully aware of their circumstances.

Participants therefore sought alternative explanations which, when fused with the dreamlike, part-sedated state, became very powerful. One explanation for the recurring idea of being trapped in the transport system is the constant activity in the ICU. For many people their normal interactions with non-stop activity, people, sounds and lights involve transport hubs such as airports, train stations, and ferry terminals. This may go some way to explaining why many patients, even those who recognise that they are critically unwell, do not make the association that they are being cared for in a clinical environment. As more than one patient mentioned in the interview transcripts, constant reminders of what is happening, where the patient is, and why they are there could help self-orientation and situational understanding.

The thematic analysis identified two recurring themes: dissociation and lack of control. This appears to reflect the inherent human fear of complete loss of autonomy. In their recreation of the patient experience of ICU Johansson *et*. *al*. describe the ICU as a “brutal and ruthless uncontrollable barrage”. They summarise their patient interviews by composing a representative sample of transcripts as a single patient experience reflective of the totality of those interviewed. This composite patient experience [61] is similar to the experiences of the interviewees who also describe feeling trapped, terrified, and alone.

Many patients found their restriction of movement and inability to perform basic activities that are taken for granted as a healthy functioning adult shocking and intolerable. Unable to sit up, eat and drink unaided, or move about at will, they describe an unsettling window to the edges of life. The indignities and shock that they experience seem to trigger a sense that they are somehow no longer civilized beings [62]. Similar, although more persistent, social stigmas and lifestyle changes enforced by debilitating illness have been described in more detail elsewhere [63, 64].

Unnerving and distressing situations that patients experience could lead to a state of heightened arousal and hyper-vigilance. On-going anxiety, also described as a persistent awareness of possible future danger [65], will increase an individual’s negative interpretation of events. Where individuals feel isolated the ‘threat belief’ is likely to flourish [66]. The narratives we analsysed confirm that many patients retain awareness whilst on the ICU.

Current guidelines indicate that lighter sedation is related to shorter durations of ventilation and intensive care, and better patient outcomes [30, 67–70]. Although research indicates that daily sedation holds do not increase the likelihood of future post-traumatic stress disorder [68, 71], in our cohort of interviewees the moments of awareness during procedures or exposure to intrusive alarms do seem to lead to patients making interpretations that can cause anxiety. Repeated reminders could help self-orientation and situational understanding.

Patients treated on ICUs have little personal control, are frequently subjected to invasive procedures, and are given a number of drugs with varying effects on consciousness and cognition. The unfamiliarity of the environment and the difficulties in communicating with staff compound their sense of disorientation and isolation. The complexity and constraints of the equipment to which the patients are attached for physiological support and monitoring, probably contribute to the feelings of persecution, kidnap, and restraint expressed by a number of patients.

Humans have been referred to as the ‘story telling ape’ [72], needing to find narrative explanation for situations and events. Patients are therefore making sense of their extraordinary circumstances by creating personal explanations that may seem irrational to others. If one considers that our identities are formed through narrative interpretation of our experiences [73], patients unable to match their vivid memories with any factual account of their experience are left unable to cope. Memories are malleable [74–76]. Those which are continually replayed, either in the mind or in conversation with others, are perhaps more susceptible to change as each cycle of repetition provides a new opportunity for modification [77, 78]. This hypothesis drives some post-traumatic stress disorder treatments designed to block the memory reconsolidation process and disrupt fear memory retention [79]. The unresolved tension between a personal narrative and the factual account may be the source of some patients’ ongoing psychosocial difficulties.

The very specific nature of ICU-acquired delirium and the transient but acute exposure to disrupted cognition could indicate a need for psychological support earlier than is traditionally offered. This could be as simple as working with the patient to cope with their anxieties whilst in the unit through early intervention strategies [80, 81], using patient diaries to maintain a detailed narrative of their stay [82] and offering opportunities for discussion in follow up clinics [83] although evidence for these is, at present, limited [84, 85].

The strength of this study lies in the breadth of first-hand experiences available in the archive. However, these data were not collected expressly to study delirium and sleep disturbances. Although a great number of such experiences were described, they were not necessarily prompted by questioning, and it is therefore possible that some patients did not share this aspect of their ICU experience during their interview. These are self-reported accounts and we cannot comment on the extent to which patients’ experiences may or may not have been exacerbated by the medications they were taking. We also cannot confirm delirium diagnoses in this cohort as patients were not asked to disclose this information during their interviews, and their medical records were not reviewed. The strength of qualitative data is that it can offer rich sources of information and insight into the lived patient experience which can offer a better understanding of a topic, in this case being critically ill and treated in an ICU. The nature of qualitative research means participants’ voices remain individual throughout the data collection, analysis, and dissemination process and offer potentially rich insights. Reporting such a study does not aim for statistical significance through a numerically representative sample. We acknowledge the limited clinical conclusions that can be drawn from these data, however the patient voice can raise awareness in the clinical community of the ideas that may lead to delirious and delusional behaviours.

Benefits of secondary analysis include a reduction in the burden of research for participants and their families, economic and resource benefits for research institutions, and long-term use of relevant data. There are some inherent limitations associated with the qualitative approach that may affect the validity of any work undertaken outside of the original study aims. In this case we were able to assess the quality of the original dataset [86] and determine that the initial data collection was rigorous and that the data could reasonably be expected to include information relevant to the new focus. Whereas original researchers have the benefit of a direct connection with the interviewees, secondary analyses are conducted solely on the interview transcripts. The researchers are at a greater distance from the data and it is likely therefore that some meta-data (for example body language, intonation, and researcher reflexivity) is lost which may affect interpretation [87]. Conversely, the distancing effect of analysis performed by a researcher outside of the original team offers validation of the initial study [86, 88, 89]. Our secondary analysis has critically assessed the evidence as presented through the verbatim transcripts and we have situated the patient experiences within a wider social context [90].

## Conclusions

Through a comprehensive review of all the interview transcripts available to us we have been able to present a compelling insight into patient experiences of the ICU which could be invaluable for staff caring for them. This paper therefore offers a description of how patients can feel during their stay on the ICU, what this might mean sociologically, and how clinical teams might approach the care of patients with suspected or confirmed delirium.

Patients and their family members are often concerned about their mental state. It is clear from their interviews that patients do not want empty reassurances that their experiences are “normal”. Instead they express the need to have their interpretation of events acknowledged; for them, these thoughts are anything but “normal”. Screening tools can offer reassurance that mental health is being monitored alongside physiological variables. Routine delirium testing also provides a structured opportunity to engage patients in their care, and to encourage them to share their thoughts and fears. The staff members working in ICU are therefore ideally positioned to recognise anxieties, and could be trained to work with patients to develop coping strategies whilst they are in the ICU. Early mobilisation, a clearly defined daily structure, repeated orientation and noise reduction have all been shown to reduce the incidence of delirium in, and outside of, the ICU [91, 92].

Most people who develop ICU-acquired delirium or experience delusions do not suffer persistent effects, but those who find it difficult to come to terms with their experiences are at risk of developing post-traumatic stress disorder, and other associated mental health problems [93]. Although many ICUs hold follow-up clinics which do identify those with longer term issues, these interviews, along with other accounts of ICU-psychosis [23], and the original exploration of these interviews which found that patients continue to feel isolated and frightened after discharge to a general ward [50], indicate that there are many more patients whose experience of the ICU could be improved with earlier support.
