# Correction: “I Can Remember Sort of Vivid People…but to Me They Were Plasticine.” Delusions on the Intensive Care Unit: What Do Patients Think Is Going On?

**DOI:** 10.1371/journal.pone.0160296

**Published:** 2016-07-25

**Authors:** Julie L. Darbyshire, Paul R. Greig, Sarah Vollam, J. Duncan Young, Lisa Hinton

There is an omission in the first sentence of the Acknowledgments section. The correct sentence is: We are grateful to the Health Experiences Research Group, Department of Primary Care Health Sciences, University of Oxford for permission to conduct a secondary analysis of interviews collected by Dr. Suman Prinhja for their study of experiences of intensive care, which was funded by ICNARC.
